# COVID-19 Pandemic: What about the Safety of Anti-Coronavirus Nanoparticles?

**DOI:** 10.3390/nano11030796

**Published:** 2021-03-19

**Authors:** Dina A. Mosselhy, Jenni Virtanen, Ravi Kant, Wei He, Mady Elbahri, Tarja Sironen

**Affiliations:** 1 Department of Virology, Faculty of Medicine, University of Helsinki, P.O. Box 21, 00014 Helsinki, Finland; jenni.me.virtanen@helsinki.fi (J.V.); ravi.kant@helsinki.fi (R.K.); tarja.sironen@helsinki.fi (T.S.); 2 Department of Veterinary Biosciences, Faculty of Veterinary Medicine, University of Helsinki, P.O. Box 66, 00014 Helsinki, Finland; 3 Nanochemistry and Nanoengineering, Department of Chemistry and Materials Science, School of Chemical Engineering, Aalto University, 02150 Espoo, Finland; mady.elbahri@aalto.fi; 4 Microbiological Unit, Fish Diseases Department, Animal Health Research Institute, Dokki, Giza 12618, Egypt; 5 School of Materials Science and Engineering, University of Science and Technology, Beijing 100083, China; hewei881130@126.com; 6 Suzhou Xiangcheng Medical Materials Science and Technology Co., Ltd., Suzhou 215123, China; 7 Nanochemistry and Nanoengineering, Institute for Materials Science, Faculty of Engineering, Kiel University, 24143 Kiel, Germany; 8 Center for Nanotechnology, Zewail City of Science and Technology, Sheikh Zayed District, Giza 12588, Egypt

**Keywords:** SARS-CoV-2, human–animal interfaces, nanoparticles, nanotoxicology, lung cell lines, zebrafish, skeptical opinion

## Abstract

Every day, new information is presented with respect to how to best combat the severe acute respiratory syndrome coronavirus 2 (SARS-CoV-2). This manuscript sheds light on such recent findings, including new co-factors (i.e., neuropilin-1) and routes (i.e., olfactory transmucosal) allowing cell entry of SARS-CoV-2 and induction of neurological symptoms, as well as the new SARS-CoV-2 variants. We highlight the SARS-CoV-2 human–animal interfaces and elaborate containment strategies using the same vaccination (i.e., nanoparticle “NP” formulations of the BNT162b2 and mRNA-1273 vaccines) for humans, minks, raccoon dogs, cats, and zoo animals. We investigate the toxicity issues of anti-CoV NPs (i.e., plasmonic NPs and quantum dots) on different levels. Namely, nano–bio interfaces (i.e., protein corona), in vitro (i.e., lung cells) and in vivo (i.e., zebrafish embryos) assessments, and impacts on humans are discussed in a narrative supported by original figures. Ultimately, we express our skeptical opinion on the comprehensive administration of such antiviral nanotheranostics, even when integrated into facemasks, because of their reported toxicities and the different NP parameters (e.g., size, shape, surface charge, and purity and chemical composition of NPs) that govern their end toxicity. We believe that more toxicity studies should be performed and be presented, clarifying the odds of the safe administration of nanotoxocological solutions and the relief of a worried public.

## 1. Introduction

### 1.1. SARS-CoV-2: Recent Discoveries

Coronaviruses (CoVs; falling under the order *Nidovirales*, family *Coronaviridae*, and subfamily *Coronavirinae*) are versatile enveloped single-stranded RNA viruses, causing respiratory, enteric, hepatic, and neurological diseases of a broad severity spectrum among different animals and humans [1]. Patients afflicted with the severe form of severe acute respiratory syndrome coronavirus 2 (SARS-CoV-2), SARS-CoV-1, and the Middle East respiratory syndrome coronavirus (MERS-CoV) share the key feature of developing acute respiratory distress syndrome (ARDS). A holistic feature associated with SARS-CoV-2 infected patients is the cytokine storm (i.e., excessive production of proinflammatory cytokines enhanced by the activated coagulation cascade), characterized by long-lived lung damage and fibrosis that may cause the low quality of life, multiorgan failure, and ultimately death [2,3]. One appealing work that shed light on a new multisystem inflammatory syndrome (MIS-C) that expressed cytokine storm (i.e., high serum IL-6 levels with a need for positive inotropic support), and was detected in SARS-CoV-2 infected older school-aged children and adolescents. The work compared the clinical features of MIS-C with Kawasaki disease (KD, a febrile children’s illness, including inflammation of the blood vessels that could cause dilatation of coronary arteries). The main differences were as follows: (i) the epidemiology of MIS-C was correlated with African descent children, and no cases were detected in China and Japan. On the contrary, Asian children demonstrated the highest KD in the world; and (ii) there was an age bias for MIS-C, with it being reported mainly in older children and adolescents. Conversely, KD occurred in children <5 years old and peaked at ~10 months old. The reasons behind the epidemiology of MIS-C, especially its absence in China (i.e., the country firstly reported SARS-CoV-2) and the contributing link of SARS-CoV-2 to the coronary dilatation in children, still need to be elucidated [4].

One team of researchers [5] unraveled the contribution of the stability of SARS-CoV-2 in aerosols and on surfaces to the induced super-spreading events, shedding light on the credibility of SARS-CoV-2 aerosol and fomite transmission. SARS-CoV-2 remained viable and infectious in aerosols for three hours and for up to three days on surfaces, with greater stability on plastic and stainless steel than on copper and cardboard. Every day, a new scientific clue is being discovered on the SARS-CoV-2. Excitingly, one group of researchers [6] recently shifted the attention from the role of angiotensin-converting enzyme 2 (ACE2) as the primary mediator behind the uptake of SARS-CoV-2 to the substantial role of other co-factors, facilitating SARS-CoV-2 cell entry independently of ACE2. Namely, neuropilin-1 (NRP1, binding furin-cleaved substrates) facilitated SARS-CoV-2 infectivity. The authors also demonstrated the blockage of this NRP1 role by a monoclonal blocking antibody against NRP1, using 80 nm Ag NPs coated with prototypic NRP1-binding CendR peptide RPARPAR_OH_. They concluded that NRP1 could be a potential target for novel antiviral drugs inhibiting SARS-CoV-2 infections. Another investigation [7] demonstrated transmucosal SARS-CoV-2 entry via regional nervous structures with olfactory tract transportation, explaining the reasons behind the associated neurological loss of smell and taste of COVID-19 patients. The study was executed on 33 fatal COVI-19 cases while investigating the olfactory mucosa, its nervous projections, and other central nervous system (CNS) regions. Viral RNA loads were detected by RT-qPCR. Interestingly, the highest SARS-CoV-2 RNA levels of SARS-CoV-2 S protein were detected in the olfactory mucosa and micro-thrombosis, and CNS infarctions were identified in 18% of cases. Another work [8] demonstrated that the SARS-CoV-2 spike protein mutation (D614G substitution) enhanced viral replication (via the increased infectivity and stability of G614 virions) in human lung epithelial cells and primary human airway tissues. Infected Syrian hamsters showed higher G614 viral titers in the upper airways (i.e., nasal washes and trachea) than in the lungs, suggesting a critical role played by D614G substitution in the enhanced viral replication in the upper respiratory tract that might propose higher viral transmissibility. Furthermore, the sera of infected hamsters demonstrated higher antibody neutralization titers against the G614 virus than the D614 virus, drawing an observation that the D614G substitution might decrease the potency of the COVID-19 vaccine candidates based on the original D614 sequence. The authors recommended further studies on the emerging SARS-CoV-2 mutations and their impact on the efficacy of vaccine candidates. 

The SARS-CoV-2 viral genome is composed of 29,903 nucleotides [9]. A research team [10] demonstrated the evolution of a drastically reduced CG content in open reading frames (ORFs) of SARS-CoV-2 with a frequency of only 1.47% (i.e., 439 CGs/29,902 nucleotides), which is much less than the expected 3.60% CG dinucleotide frequency in the viral genome. Based on the energy usage bases, a coronavirus with low CG content translates its RNA more efficiently. This efficiency stems from the lower energy required to disrupt stem-loops formed in the viral genome’s secondary structure, meaning less stable ORFs that do not adversely recruit host ribosomes to start viral RNA translation. Consequently, the team claimed that SARS-CoV-2 was less virulent than the MERS-CoV in the highly stable ORFs.

### 1.2. SARS-CoV-2: Human–Animal Interfaces

One cause of concern and speculation since the beginning of the pandemic has been virus transmission between animals and humans. Supposedly, this transmission could lead to virus reservoirs among companion animals and wildlife and new vaccine-resistant mutations, which would greatly hinder the efforts to control the pandemic. Since spring 2020, SARS-CoV-2 outbreaks have been reported in mink farms in several countries, including signs of both human-to-mink and mink-to-human transmission [11]. These transmission signs triggered the European Centre for Disease Prevention and Control [12] and the WHO to highlight the importance of surveying the human–animal interface and collaboration among virologists and epidemiologists to tackle viral mutations. Specifically, farms, where animals live in crowded conditions, could be ideal for efficient, rapid viral spread and mutation formation. The viral RNA has been detected in airborne inhalable dust of mink farms, leading to speculation that dust may be a means of transmission among minks, as well as raising concerns about human occupational exposure. Despite the best efforts to prevent SARS-CoV-2 spread, the virus has spread widely on mink farms in the Netherlands (i.e., in North Brabant Noord Brabant, and Limburg provinces) [13] and Denmark, with detections also reported in Spain, France, Italy, Sweden, Greece, Canada, and Lithuania (reported as of the 4th of January 2021 by the OIE-World Organization for Animal Health [14]). The number of culled mink in the Netherlands during the outbreak has exceeded 2.7 million animals, which is much more (6.5 times) than the number of confirmed human cases [15]. SARS-CoV-2 has also been reported in mink from Poland (i.e., the second-largest producer of mink pelts in Europe, following Denmark, with 15 positive cases at a farm in Northern Poland) [16]. The USA is also a leading mink producer, and considering the surge of reported SARS-CoV-2 infected human individuals in these countries (i.e., the USA, Denmark, Poland, the Netherlands, and Canada) linked with specific high mink densities, it is strongly speculated that there are possible unreported SARS-CoV-2 infections in commercial fur farms [17]. 

When three farms in Northern Jutland in Denmark reported SARS-CoV-2 infected mink individuals, all SARS-CoV-2 RNA mink sequences showed a mutation (nt C25936T [as cDNA] encoding H182 to Y within ORF3a) that was also found in the associated human individuals. Despite the absence of this mutation in human SARS-CoV-2 Jutland sequences before 10 June 2020, it hit more than 40% frequency between 10 June and 1 July 2020. Moreover, this mutation was found in a NB03 mink farm in the Netherlands [18]. Another mutation was detected in the spike gene (A22920T, encoding Y453 to F) in these three Danish farms and a farm in the Netherlands. In the same vein, this mutation was not present in humans before June 10, but later on, was detected in humans linked to infected farms in Denmark. One more mutation in the ORF1b gene (C15656T, encoding T730 to I) was only detected in mink-human sequences from Denmark and a sequence from New Zealand [18]. The Danish health authorities then announced a saddening mink culling (i.e., 17 million animals) following the rapid uncontrolled spread of SARS-CoV-2 in mink farms (i.e., more than 200 farms since June), mink-associated mutations, and human cases infected with mink-related variants in regions of such mink farms. What raised alarm bells were the following: (i) the viral mutations (i.e., Cluster-5 variant causing two deletions in the spike protein) in samples collected from mink and infected people; and (ii) the suggested decreased ability of antibodies of recovered human cases to neutralize the Cluster-5 variants, triggering the concern that the variant could make the vaccines less effective, even though this Cluster-5 variant almost stopped spreading and had not been detected since September [19]. 

Regarding SARS-CoV-2 infections in other animals, a new work [20] identified the susceptibility of raccoon dogs to SARS-CoV-2 infection and the ability of raccoons to transmit the virus to directly to animals they come in contact with. A recent review [21] investigated the critical role of animals as reservoirs, natural hosts, and experimental models for SARS-CoV-2. Regarding reservoir animals, there is some consensus on the evolution of SARS-CoV-2 following multiple “mosaic” recombination events of the bat and pangolin SARS-related CoVs (SARSr-CoVs) due to the 96% genetic similarity of SARS-CoV-2 and horseshoe bat SARSr-CoV (designated RaTG13). Concerning natural infections of animals, the review reported the following: (i) dogs (in Hong Kong and Germany) were identified positive for SARS-CoV-2 with speculation of human-to-dog transmission from the infected owners; (ii) cats in China, Hong Kong, USA, Belgium, have been found to be positive for SARS-CoV-2, suggesting human-to-cat transmission, with cats having a greater susceptibility to SARS-CoV-2 than dogs; and (iii) tigers and lions in zoos (in the USA) were confirmed to be positive for SARS-CoV-2, with infection assumed to be from an asymptomatic infected zookeeper. Experimental animal models, monkeys, hamsters, ferrets, cats, tree shrews, transgenic mice, and fruit bats are regarded as appropriate animal models for studying the transmission and pathogenesis of viral infection and replication. Moreover, monkeys (i.e., rhesus macaques are considered to be a suitable animal model for investigating vaccine efficacy against SARS-CoV-2, in a similar fashion to their use in SARS-CoV and MERS-CoV [21]. 

### 1.3. Repurposed Drugs and Vaccines against SARS-CoV-2

A futile question that will require a couple of years before it can be answered is “what is the specific antiviral drug that could combat the COVID-19?” Instead, we could ask more realistic questions, such as “what are the potential repurposed drugs that could solve the current pandemic node?” A recent work [2] mentioned a couple of host-directed therapies, including metformin, glitazones, fibrates, sartans, and atorvastin to boost the immune response preventing ARD and zinc formulations possessing some antiviral properties, as adjuvants with antiviral therapies targeting COVID-19. Moreover, cellular therapy using allogeneic mesenchymal stromal cells reduced non-productive inflammation and laid the ground for a new therapy path under clinical trials (phase I/II) for ARD patients. Additionally, prophylactic doses of low-molecular-weight heparin are the mainstay in preventing venous thromboembolism in hospitalized COVID-19 patients. Drugs antagonizing thrombin (i.e., a protease serve clot formation) receptors could also play a promising role in mitigating the severity and bad prognosis of COVID-19 [3]. Based on the preliminary results of a RECOVERY trial, a low dose (6 mg once daily for ten days) of dexamethasone, dubbed ‘a major breakthrough’, reduced deaths by one-third in hospitalized COVID-19 patients under ventilation (2104 patients) and by one-fifth in hospitalized patients under standard healthcare (4321 patients) [22]. Dexamethasone is a glucocorticoid with anti-inflammatory effects mimicking those of the natural hormones (i.e., cortisol in humans and corticosterone in rodents), inhibiting the release of inflammatory chemokines and reducing lung inflammation and ARDS severity [23,24]. Attention has been directed towards the use of psychotropic drugs (e.g., nicotine, the anti-psychotic aripiprazole, and the anti-depressant sertraline) preventing SARS-CoV-2 infections after noticing the conflicting fact of almost empty COVID-19 units in the psychiatric clinics in France. This conflict stemmed from the thought that psychiatric clinics would be a focal point for disseminating the infection due to the non-adherence of psychiatric patients to protective measures and their late seeking of medical help, as they experience social discrimination. These psychotropic drugs are cationic amphiphilic drugs (CADs), disturbing intracellular trafficking. Even though nicotine is a partial CAD, it could specifically bind to specific receptors (e.g., acetylcholine, angiotensin-converting enzyme 2, and sigma-1), preventing SARS-CoV-2 cellular entry and inducing different molecular consequences [25]. A group of researchers [26] showed an antiviral effect of the other repurposed anti-parasitic ivermectin against SARS-CoV-2 in vitro (i.e., in the infected Vero/hSLAM cells), reducing the viral replication by ~5000 fold in SARS-CoV-2 RNA analyzed by RT-PCR within 24 to 48 h. The researchers speculated the possible mechanism behind this antiviral action to be the binding of ivermectin to the importin α/β1 heterodimer, preventing its binding to the coronavirus protein and consequently preventing the viral protein entry to the nucleus. This ivermectin viral inhibition could be correlated with previous inhibitions demonstrated against other RNA viruses (i.e., non-structural protein 5 inhibition of dengue virus serotypes 1 and 2 by the ivermectin blockage to nuclear transporter importin α/β, in vitro) [27].

The world was breathing a sigh of relief with the emergence of some vaccine candidates against SARS-CoV-2 when the worrying news came from England (on December 13, 2020, from south-eastern England, Wales, and Scotland) about a new variant of SARS-CoV-2 coined VUI-202012/01 (i.e., the first “Variant Under Investigation” in December 2020). This VUI-202012/01 (20B/501Y.V1) variant has undergone 17 mutations. N501Y (i.e., a mutation in the spike protein) was defined as one of the most critical mutations, rendering the virus more infectious and more highly spreading. However, confirmation that the variant was the causative agent of the rise in such cases could not be drawn yet. On December 21, 2020, the Public Health England [28] released the exact number of 23 mutations, namely 13 non-synonymous mutations (i.e., a series of spike protein mutations and a stop codon mutation in ORF8), four amino acid deletions, and six synonymous mutations (i.e., with five in ORF1ab as C913T, C5986T, C14676T, C15279T, and C16176T, and one in the membrane gene as T26801C). Still, a recent study [29] updated the number of mutations to 24, including 14 non-synonymous mutations with the N501Y mutation in the receptor-binding domain (RBD) of the spike protein, still being recognized as an alarming mutation. The study estimated that this 501Y lineage with Δ69/Δ70 amino acid deletion that began spreading in the UK from late September and which predominated in December 2020 (i.e., 501Y Variant 2) was 75% (i.e., 70 to 80%) more transmissible than the 501N lineage [29]. Meanwhile, the earlier 501Y lineage without amino acid deletion Δ69/Δ70 (i.e., 501Y Variant 1) that circulated mainly in the UK from early September to mid-November was only 10% (i.e., 6 to 13%) more transmissible than the 501N lineage. Similarly, another 501Y lineage (i.e., 20C/501Y.V2 with Δ69/Δ70 amino acid deletion) with critical mutations in the RBD, namely, K417N, E484K, and N501Y, detected in South Africa, spread from October to November 2020 in Eastern Cape Province [30], and remained genetically distant from the UK 501Y Variant 2 [29]. This South African variant spread widely, circulating in both Eastern and Western Cape Provinces [30], with its two mutations (i.e., E484K and N501Y) in the receptor-binding motif that mainly forms the interface with the human ACE2 receptor. On the one hand, SARS-CoV-2 is an RNA virus that mutates naturally during replication, and the leading vaccine candidates target the spike protein, which has already undergone mutations. On the other hand, vaccines provoke antibody production against several regions in the spike protein. Therefore, the chance of a single change of spike protein causing a vaccine’s ineffectiveness remains low [31].

### 1.4. Nanotheranostics: Swaying from Anti-Coronavirus to Toxicological Properties

**Figure 1:**
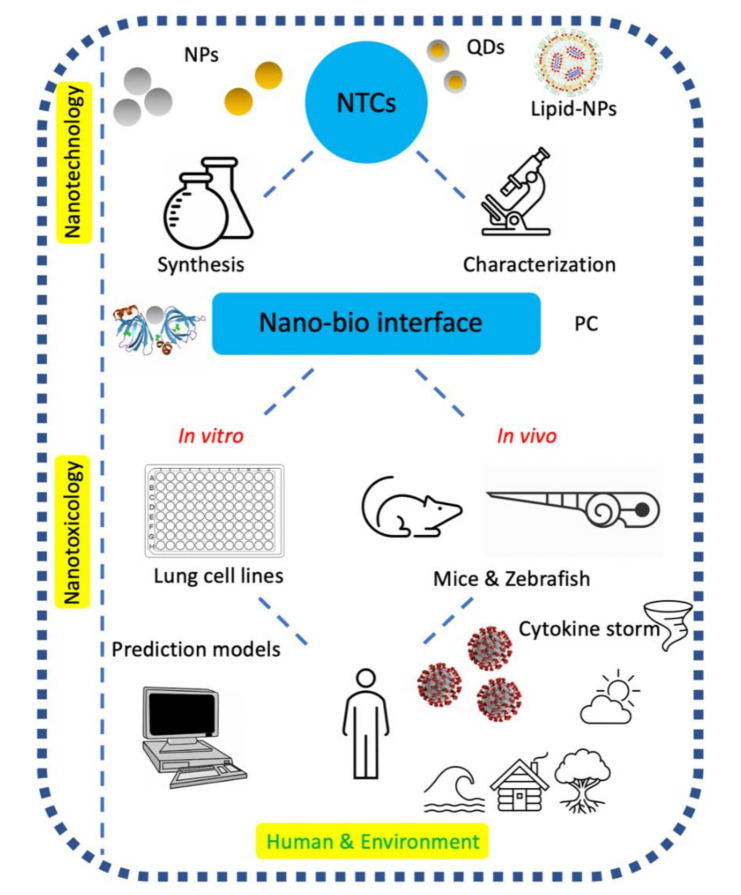
The nanotoxicology of anti-coronavirus nanotheranostics (NTCs) connects the pieces (i.e., the chemistry of nanomaterials, interfaces, and biology) of the scientific jigsaw puzzle played by humans in our shared environment in attempting to decipher their safety issues.

The term “theranostics” refers to the combination of diagnostic and therapeutic properties within a single agent. Using the advantages of nanotechnology to refine the term into nanotheranostics as an “all-in-one approach” facilitates precision medicine [32,33]. An intriguing property of NPs is that they share the nanometer size distribution with viruses. Consequently, NPs can enter virus-targeted cells in a similar way to viruses [34]. Nanotechnology could be appealing in vaccine design, since NPs could be ideally utilized as carriers for antigen delivery, adjuvants, and mimicking agents for viral structures [35]. On 18 November 2020, the promising news of SARS-CoV-2 prophylaxis came from the BioNtech and Pfizer companies, declaring the results of their COVID-19 vaccine (i.e., BNT162b2) phase 3 clinical trial. This news came directly after Moderna announced the preliminary outcomes of their phase 3 study for the other vaccine (i.e., mRNA-1273), developed by the Cambridge-based biotech company in collaboration with the National Institutes of Health. A characteristic property of several mRNA-based therapeutics, including BNT162b2 and mRNA-1273, is the use of lipid NPs as the first-line vehicles, facilitating endosomal escape after their endocytosis and the release of their genetic cargo in the cytosol. The mRNA is then translated into antigenic proteins (i.e., SARS-CoV-2 spike protein), promoting the neutralization of antibody production by the immune system. Collectively, these lipid nanocarriers would serve three essential purposes, namely, (i) maintenance of mRNA conformation and stability, (ii) protection of mRNA cargos from degradation via the endosomal escape, and (iii) efficient cellular uptake via the targeted mRNA delivery [34]. Even though BNT162b2 and mRNA-1273 would not be the first approved nanoformulations for human use, the odds surrounding nanomedicine and the nano–bio interactions remain understudied [36]. Previous reports have demonstrated the crucial role of some nanotheranostic systems in rapid (i.e., 10 min) CoV diagnostics using Ag NPs [37] and Au NPs [38] and efficient in vitro CoV inhibitory effects using carbon dots [39,40] and Ag_2_S QDs [41]. A primary concern with nanotheranostics is human exposure; for example, the exposure of researchers synthesizing the nanoparticles (NPs) and of consumers of products containing NPs. There are several routes of exposure, dictating the trajectory and fate of NPs [42]. Unfortunately, carrying out research directed towards understanding the fate of NPs (in both humans and environments) and preventing the risks associated with them remains highly unappealing in the highly competitive worlds of intellectual property, research funding, and technology development [43] (i.e., anti-coronavirus NP formulations). Nanotoxicology is a branch of science dealing with the interactions of nanoparticles with biological systems, mapping the connections and interfaces between chemistry (size, shape, composition, aggregation, surface charge, and surface functionalities of NPs) and biology (induced toxic effects) [42] (as seen in Figure 1). While nanotoxicity research has been flourished on in vitro cell culture models, there is still very little scientific understanding of in vivo assessments of NPs because in vivo models are more complex and sophisticated [44]. Rodents (mice and rats) and rabbits are popular animal vertebrate models employed in toxicological studies because of their cheap costs and wealth of available information on their growth, reproduction, and toxicology corresponding to humans. However, the hurdle remains in the slow embryonic development of rodents and rabbits. Zebrafish (*Danio rerio*) embryos cross this hurdle as an animal model full of promise due to (i) their short life cycle, (ii) embryo transparency, facilitating a reliable observation of toxic effects, (iii) molecular accessibility for gene manipulation, constituting a homologous 85% of protein-coding genes to human counterparts, and (iv) their short reproduction cycle and high fecundity, enabling one female to produce 100 to 300 embryos. Despite the extensive antibacterial administration of Ag NPs, their toxicological exploitation in zebrafish still in an early stage [45]. A further in vivo complication lies in the fact that NP/biological interactions and interfaces provoke various biocompatible or biodiverse effects in humans and the environment concerning the distribution, metabolism, immune response, and excretion of NPs [42].

With respect to more specific toxicities related to NPs, subchronically inflamed human small airway epithelial cells exposed to metal oxide and copier center NPs are susceptible to pneumonic nanotoxicity [44], which could be related to and may have contributed to the COVID-19 pandemic, causing pneumonia, especially in immunocompromised patients [46]. Inhalation of high levels of NPs (possessing ultra-high surface-to-volume ratios and deposition rates in the lung alveoli) leads to a risk of developing respiratory and cardiac diseases besides lung cancer [47]. The toxicity issues surrounding quantum dots (QDs) are a disputed subject regarding their biomedical applications. These toxicity issues and beliefs emerge from the toxicity of cadmium-containing QDs towards cultured cells, with their toxicity towards humans and in all of their other forms being extrapolated on the basis their being homogeneous materials. However, this extrapolation is refuted by the lack of studies demonstrating such toxicities in animal models and the heterogeneity of the unique physicochemical properties of various QDs [48]. Metal NPs ending in the aquatic environment could be magnified in aquatic organisms such as fish through the food chain. After human consumption of water or organisms containing these NPs, including vegetables, fish, and livestock, the metal NPs could eventually accumulate in the human body, posing a threat to human health [45]. Despite this cumulative threat, research investigating the toxic mechanisms of metal NPs in zebrafish (as an aquatic vertebrate model) remains unsatisfactory [45]. 

Consequently, this work will generate fresh insight into safety concerns (in vitro, in vivo, and with respect to human and environmental exposures) and nano–bio interfaces of anti-CoV nanotheranostics (as displayed in Figure 1). We mainly address the safety issues concerning plasmonic NPs and QDs, which were discussed as promising antiviral nanotheranostic systems in our previously accepted review article [49], investigating the mechanistic actions of diagnosis and treatment with nanotheranostics in combating coronavirus infections. Other significant areas where this study makes original contributions include: (i) garnering and interpreting the recent facts explored regarding SARS-CoV-2; (ii) highlighting the SARS-CoV-2 human–animal interfaces that have been missing in literature; and (iii) exploring the realistic options of repurposed drugs and vaccines against SARS-CoV-2. We finally pinpoint the challenges associated with implementing anti-CoV nanotheranostics and our own inputs with respect to the next steps in this flourishing field of anti-CoV NPs.

## 2. Nano–Bio Interface

The unusual properties of NPs have a significant impact on the structure and function of proteins, making it intriguing to advance the understanding of such effects and interfaces [50]. Therefore, it is fitting first to ask what is meant by nano–bio interfaces? Nano–bio interfaces encompass the dynamic physicochemical interactions, kinetics, and thermodynamic exchanges between the surfaces of NPs and biological components (e.g., proteins, phospholipids, membranes, membrane-bound vesicles, organelles, DNA, biological fluids, and urine) [51,52]. The nano–bio interface (as depicted in Figure 1) is best described as comprising three interacting components: (i) the surface of NPs (characteristics dictated by the physicochemical composition of NPs); (ii) the solid–liquid interface and the changes associated with the interactions between NPs and the surrounding media; and (iii) the contact zone of the solid–liquid interface with biological substrates [51].

The penetration of NPs into the biological fluid (e.g., blood, plasma, or interstitial fluid) shapes a protein corona (PC). The characters of the PC formed would not only depend on the physicochemical properties of NPs (i.e., size, shape, composition, surface functional groups, and surface charges) and the biophysical properties of the biological media (i.e., blood, interstitial fluid, or cell cytoplasm), but also on the protein–protein interactions due to their competition to adsorb on the highly reactive NP surfaces [53]. NPs are decorated and coated with proteins, propelled by a potential energy gradient that could undertake conformational changes. These changes expose new epitopes or altering functions. Consequently, the PC frames a new biological identity for the NPs (e.g., surface properties, charges, resistance to aggregation, hydrodynamic size, and composition of PC) [51,52,54]. The life spans of NP–ligand complexes last from microseconds to days, influenced by the protein concentrations in the vicinity of NPs, which differ from one biological compartment to another [51]. Environmental factors, including temperature, local heating, and incubation time, also influence PC composition [52]. However, the specific time until which NP–ligand complexes exhibit biological significance remains, unfortunately, a mystery [54]. Some tricky attempts could still be made to make sense of this particular binding time, such as using a fixed quenching agent on the protein. Or the protein adsorption to the NP could be traced via fluorescence decay [54]. The PC architecture may be divided into (i) hard PC, representing the strong NP–protein interactions achieved within seconds to minutes, and which is the closest layer to the surface of the NPs; and (ii) soft PC, representing the loosely bound external layer of protein–protein interactions achieved within hours [53,55]. Furthermore, the soft corona proteins could also interact with the hard corona proteins during their desorption from NP surfaces and empty a site for other biomolecular interactions. These hard and soft PCs describe the nano–bio interface’s dynamic nature between NPs and proteins. This dynamic nano–bio interface is governed by a series of interactions, namely hydrodynamic, electrostatic, electrodynamic, solvent, and steric interactions. Such interactions can be explained as follows: (i) hydrodynamic interactions, representing long-range (i.e., within 10^2^ to 10^6^ nm range) interactions between particles moving within a viscous fluid, transport, shear, lift, and Brownian diffusions, increasing the collision between NPs and other surfaces in the system; (ii) electrostatic interactions, which are Coulomb interactions (within 1 to 100 nm range) between charged interfaces and co-ions, forming an electrostatic double layer; (iii) electrodynamic interactions (within 1 to 100 nm range), which are attractive Van der Waals interactions; (iv) solvent interactions (within the smallest range of 10 to 10 nm), which are those including lyophobic or lyophilic materials and solvent molecules; and (v) steric interactions, which are those repulsive interactions (within 1 to 100 nm range) with other interfaces resulting from the adsorbed polymers, increasing the stability of individual NPs, while interfering in cellular uptake [53]. 

**Figure 2:**
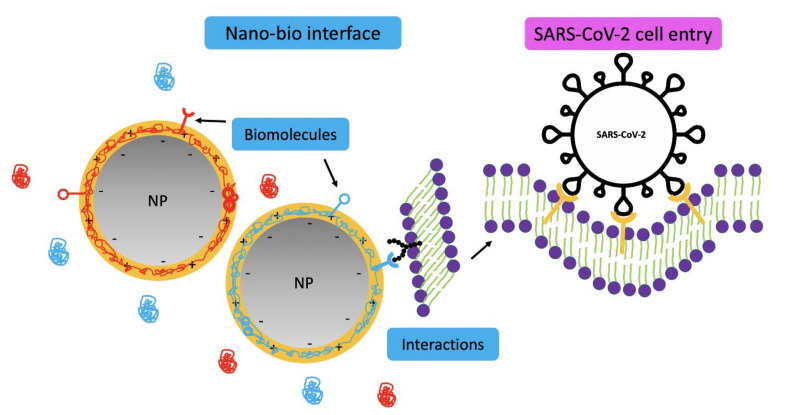
Interactions and forces between nanoparticles (NPs) and biological interfaces (i.e., protein corona, PC) with an accompanying illustration of cellular endocytosis of SARS-CoV-2, which is also spherical and falls within the nanometer range. Interactions and forces that occur at the contact of NPs with cells or upon suspension in biological media include biomolecular interactions, repulsive electrostatic forces, attractive Van der Waals and depletion forces, and covalent forces [51].

A critical general role of the PC is to tag NPs in order for them to be more recognizable by innate immunity, quickly clearing the NPs via the phagocytic cells in the lungs, liver, and spleen. Consequently, PCs express NP immunogenicity [55]. NP–membrane wrapping describes the adhesion and engulfment of NPs in a cell surface lipid bilayer, which can be an advantage for therapeutic drug delivery purposes. This wrapping is governed by specific (ligand–receptor, facilitating receptor-mediated endocytosis) and nonspecific binding interactions guided by three prime characteristics of NPs (as displayed in Figure 2). These are, namely, (i) surface charge, playing a crucial role in the interactions of NPs with charged phospholipid groups or protein domains on cell surfaces, considering the more robust effects of cationic surfaces than their anionic counterparts; (ii) hydrophobicity, where hydrophobic NPs are more rapidly engulfed than their less hydrophobic counterparts, because more hydrophobic surfaces are more reliable for aggregation, facilitating their early removal by the reticuloendothelial system; and (iii) roughness, where the smaller NP surface protrusions or depressions decrease repulsive interactions and enhance adhesion and cellular uptake [51]. Deng and colleagues [56] demonstrated that the negatively charged poly(acrylic acid)-conjugated gold NPs (GNPs, of size 5 nm) bind to specific sites on fibrinogen (length of ~45 nm and diameter of 5 nm) and induce fibrinogen unfolding. This unfolding is proinflammatory and provokes an integrin receptor (Mac-1) interaction. Mac-1 activation increases the NF-kB (nuclear factor kappa-light-chain-enhancer of activated B cells) signaling pathway and elegantly releases inflammatory cytokines in an alternative mechanism rather than the traditional understood oxidative stress.

**Figure 3:**
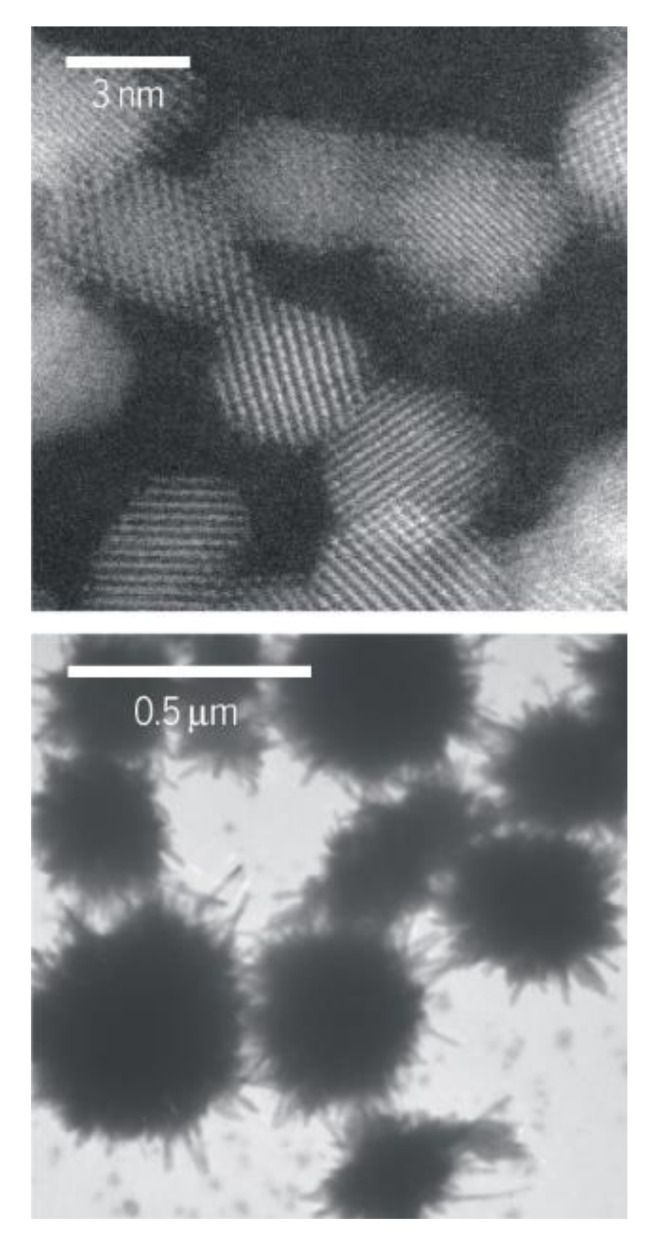
Aging of ceria nanoparticles (NPs). Different holistic properties of young (upper) and aged (lower) ceria NPs after being released into the environment. Reprinted with permission from ref. [57]. Copyright, 2015, The American Association for the Advancement of Science.

With respect to the nano–bio interface, it is vital to uncover the implications of the ‘Trojan-horse’ effect and the ‘aging’ of NPs. The ‘Trojan-horse’ effect is the active entrance and internalization of small NPs into cells and organisms after unwitting recognition by cell receptors, inducing toxicity [57,58]. The ‘aging’ of NPs is a new term describing the holistic distinctive structural and chemical properties of aged NPs compared with their pristine counterparts (as depicted in Figure 3). Regrettably, most studies in the nanotoxicology field have only focused on pristine NPs, making it useless to correlate the toxicological and environmental findings of pristine NPs to the aged ones [57].

## 3. In Vitro and In Vivo Biocompatibility Assessment of NPs

Plasmonic NPs are heavily employed for their promising surface plasmon resonance (SPR) properties, allowing early plasmonic diagnostics and enhanced therapeutics. However, pure Ag NPs could be toxic, while also being easily aggregated due to their high surface energy. Consequently, the surface functionalization of Ag NPs is a dominant safety issue. Regarding Au NPs, biocompatibility is one of their intriguing properties, facilitating wide applications, with colloidal particles ranging from 3 to 100 nm being acceptably non-toxic in vitro, while Au NPs of 1 to 2 nm could constitute hazardous effects [59]. Au NPs are also biocompatible, as long as no charge is present, while positively or negatively charged Au NPs induce toxicities [60]. Furthermore, QDs and are notable examples of NPs showing no explicit biodegradation in vivo [42]. Therefore, this section investigates the biocompatibility issues of mainly plasmonic NPs and QDs, and the factors influencing them.

### 3.1. Assessment In Vitro

NP–cellular interaction affects the genomics, proteomics, and metabolomics of cells, which could cause toxicities, resulting in apoptosis, necrosis, or autophagy. Different NP toxicities fall under the following mechanisms: (i) plasma membrane damage disrupting the permeability of ions; (ii) cytoskeleton modification reducing cellular proliferation and motility; (iii) mitochondrial toxicity increasing mitochondrial membrane permeability and inducing oxidative stress; (iv) nuclear damage, mainly executed by NPs < 5 nm, which can accumulate within the nucleus, interfering with cellular division; (v) reactive oxygen species (ROS) generation inducing oxidative stress; and (vi) interference in signaling pathways, including mitogen-activated protein kinase, NF-κB (nuclear factor kappa-light-chain-enhancer of activated B cells), bone morphogenic protein, and transforming growth factor β, causing toxicity [61]. As the primary focus is integrating anti-coronavirus nanotheranostic systems in facemasks, this section aims to clarify the consequences of accidentally inhaling loose NPs from the masks into the lungs or the impact of occupational exposure to such NPs on the lung cell lines as an investigation model.

With respect to the question of plasmonic NPs, Ng et al. [62] identified the effects of Au NPs (20 nm) treating small airway epithelial cells (SAECs) on unexposed neighboring MRC5 lung fibroblasts, inducing changes in protein expression in a co-culture system, imitating the respiratory tract. This change was translated as a significant downregulation of cell migration promoting proteins (e.g., plasminogen activator, urokinase, and chemokine growth-regulated oncogene) and increased cell adhesion enhancing proteins (e.g., paxillin, breast cancer anti-estrogen resistance 1 and caveolin-1). These protein alterations caused phenotypic changes in lung fibroblasts (i.e., cytoskeleton remodeling and increased cell adhesion), affecting lung function. In contrast to the adverse effects of Au NP previously described on lung cells, Brandenberger et al. [63] detected no adverse effects of Au NPs (15 nm) in a triple cell co-culture system, mimicking the alveolar lung epithelium, after aerosol exposure at the air–liquid interface. The authors observed the homogeneous deposition and uptake of Au NPs into the cells without mRNA induction (i.e., of proinflammatory markers, TNFα, IL-8, and inducible nitric oxide synthase, and oxidative stress markers, hemeoxygenase-1, and superoxide dismutase 2). They ultimately recommended broader chronic investigations using in vivo animal models to confirm the absence of adverse effects before entering the nanomedicine market for various applications. In the case of Ag NPs, Suliman et al. [64] investigated the toxicity of Ag NPs (56 nm) in human lung epithelial (A549) cells. Ag NPs caused dose- and time-dependent cytotoxicity in A549 cells, as shown by MTT [3-(4,5-dimethylthiazol-2-yl)22,5 diphenyl tetrazolium bromide] assay and the lactate dehydrogenase enzyme. Ag NPs induced dose- and time-dependent changes in the following: (i) cell morphology, and (ii) oxidative stress via the depletion of glutathione (i.e., antioxidant and the frontline of the cellular defense mechanism) and elevated levels of lipid peroxides, superoxide dismutase, and catalase (i.e., antioxidant enzymes), forming DNA adducts. The authors finally argued that Ag NPs induced cytotoxicity and immunotoxicity facilitated mainly by ROS generation and oxidative stress. They ultimately drew a note of caution with respect to industrial applications of Ag NPs. Two further studies verified that biosynthesized Ag NPs showed dose-dependent cytotoxicity against A549 cells as determined by MTT assay [65,66]. Gliga et al. [67] demonstrated that small Ag NPs (10 nm) displayed a noticeable cytotoxic impact on human lung cells (BEAS-2B) through intracellular Ag release. Ag NPs induced DNA damage with neither γH2AX foci formation nor increased ROS production in BEAS-2B cells. Hamilton et al. [68] also proved that smaller Ag NPs (20 nm), regardless of coating, showed greater toxicity in lung epithelial cell lines than larger Ag NPs (110 nm). The toxicity was dependent on the rate of particle dissolution, with the more enhanced and faster dissolution of smaller Ag NPs eliciting more toxicity. 

**Figure 4:**
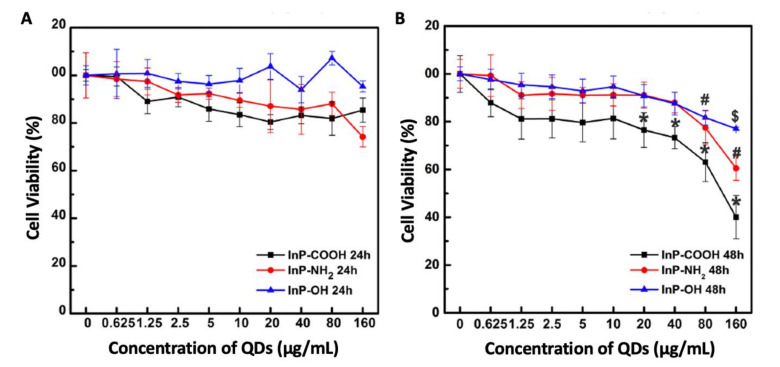
Cell viabilities of RLE-6TN cells after treatment with quantum dots (QDs). Namely, InP/ZnS-COOH, InP/ZnS-NH_2_, and InP/ZnS-OH QDs for 24 (**A**) and 48 h (**B**). Adapted from [69] under the terms of the Creative Commons Attribution License (CC BY); Frontiers Media SA, 2018.

Considering QDs as anti-CoV candidates, Chen and colleagues [69] showed the cytotoxicity of indium phosphide/zinc sulfate (InP/ZnS) QDs (with different surface groups of COOH, NH_2_, and OH and with hydrodynamic diameters of 9, 12, and 98 nm, respectively) towards alveolar type II epithelial cells, RLE-6TN. Increasing the concentrations of InP/ZnS QDs decreased the cell viability after 48 h, with the highest toxicity being recorded for InP/ZnS-COOH QDs followed by InP/ZnS-NH2 QDs, and finally InP/ZnSOH QDs (as shown in Figure 4). All InP/ZnS QDs promoted cell apoptosis after 48 h and increased ROS levels and oxidative stress after being internalized within the cells. This induced apoptosis and oxidative stress implies that caution should be exercised when adjusting the administered concentrations and surface functional groups of InP/ZnS QDs in theranostics. These results differ from those of Buz and co-workers [70]. The latter showed the absence of toxicity of N-acetyl-L-cysteine (antioxidant)-Ag_2_S QDs (with a size of 1 to 5 nm and strong emission between 748 and 840 nm, as well as sufficient stability in biological media) towards BEAS-2B (human bronchial epithelial cells) even with the high internalization, implying their cytocompatibility [70].

### 3.2. Assessment In Vivo

In the current work, we aim to thoroughly investigate the in vivo toxicity of promising anti-coronavirus nanotheranostics on different animal models, including rats, mice, and zebrafish embryos (i.e., the prime focus of in vivo toxicity assessment of the present review article). Starting the assessment with rats and mice, serum amyloid A (SAA) and C-reactive protein (CRP, a nonspecific acute-phase protein produced by hepatocytes of 0.8 mg/L concentration in normal individuals, with higher levels associated with inflammation and tissue damage [71]) levels are predictors of risk of coronary heart disease. However, rats do not express *Saa* genes, and mice only moderately express *Crp* genes [47]. The architecture of the mouse trachea is similar to its human counterpart. The mouse airways and the smallest bronchioles of humans are covered by cuboidal epithelium, lacking basal cells and containing ciliated, secretory, and neuroendocrine cells. The alveoli of mice and humans share a similar composition with two functionally distinct cell types: (i) flat and extended alveolar type I (AT-I) cells allow gas exchange; and (ii) cuboidal alveolar type II (AT-II) cells allow surfactant protein production and secretion [72]. Lin et al. [73] examined the acute toxicity of InP/ZnS QDs (with different surface modifications, including COOH, NH_2_, and OH, and hydrodynamic diameters of 10, 14, and 104 nm, respectively) in mice after pulmonary aerosol inhalation at a dosage of 2.5 mg/kg or 25 mg/kg. InP/ZnS QDs entered circulation and accumulated in the lungs and kidneys. This entrance and accumulation caused (i) immunological response via decreased lymphocyte and increased granulocyte counts; (ii) labeling of lung tissue and hyperemia in alveolar septa (i.e., lung injury, especially for the QDs with NH_2_ surface groups); and (iii) decrease in alkaline phosphatase and globulin, indicating the accumulation of adverse effects in the interference of organ functions. The authors again advised exercising caution in adjusting the surface modifications of QDs to reduce their toxicities in vivo in biological applications.

Let us connect the factors influencing the toxicity of NPs in zebrafish embryos within a single storyline by answering the following questions. Would the size of NPs govern their toxicity in zebrafish? Liu et al. [74] demonstrated the size-dependent toxicity of NPs using the same surfactant (PVP). The smaller Ag NPs (20 nm) were more toxic to zebrafish after 96 h exposure than larger ones (100 nm). This greater toxicity was determined on the basis of a decrease in the 96-h LC_50_ (median lethal concentration) from 1.34 to 2.57 mg/L. They explained the greater toxicity as being a result of the greater ability of smaller Ag NPs to enter cells compared to the aggregated larger ones. The authors demonstrated this greater toxicity on the molecular level by showing the higher expression levels of nine genes specifically expressed for gills (first entrance point of Ag NPs to zebrafish), intestines (digestive and absorptive point to Ag NPs), and muscles with the smaller Ag Nps compared to the larger ones. Another explanation for the size-dependent toxicity of Ag NPs is related to the fact that smaller Ag NPs have the ability to diffuse more into the embryos than larger particles because of the inverse correlation of the diffusion coefficient of single Ag NPs and their size [75]. A further reason for the greater toxicity of smaller Ag NPs (20 nm) compared to larger Ag NPs (110 nm) in zebrafish embryos was attributed to the particle dispersion and the release of Ag^+^ ions, which goven the toxicity of Ag NPs [76], while smaller Ag NPs release more Ag^+^ ions more quickly [77,78].

**Figure 5:**
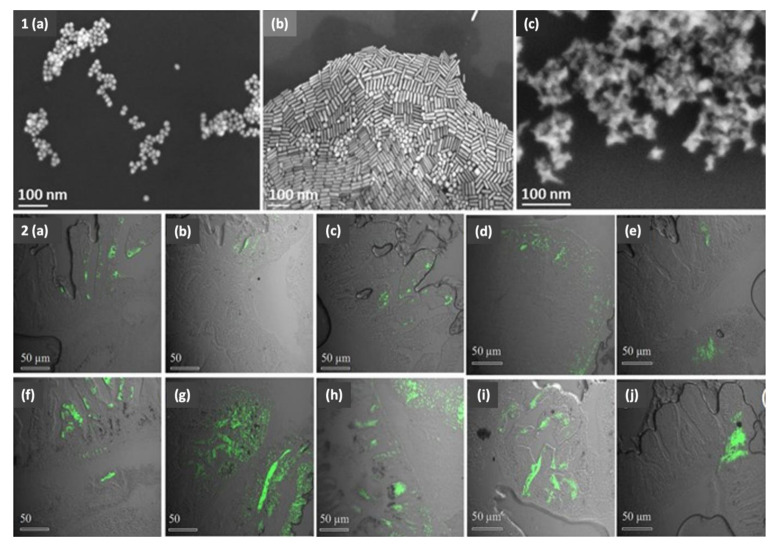
SEM images (**1**) of different shapes of Au NPs. Namely, spherical Au NPs (**a**), rod Au NPs (**b**), and star Au NPs (**c**). Confocal images (**2**) of zebrafish digestive system after injection with Fluorescein-conjugated glyco-Au NPs after different time points. Namely, spherical Au NPs after 4 h (**a**,**f**) (with slightly different sizes); rod Au NPs after 4 h (**b**,**g**) (with slightly different sizes), 24 h (**d**), and 48 h (**e**); and star Au NPs after 4 h (**c**,**h**) (with slightly different sizes), 24 h (**i**), and 48 h (**j**). Reproduced from [81] under the terms of the Creative Commons Attribution 4.0 License with modifications; Springer Nature Limited, 2017.

Turning now to the NP shape factor, what would its toxic influence be on zebrafish? Generally speaking, spherical NPs are more efficiently internalized by cells than rod-shaped or filamentous particles because of the ease with which the cellular membrane wraps around spherical NPs, decreasing their circulation time in vivo [61]. Explicitly speaking, Abramenko et al. [79] demonstrated the greater toxicity (lower LC_50_) of Ag nanoplates (0.0415 LC_50_ with a size of 28 nm in the egg water) than that of Ag nanospheres (0.0169 LC_50_ with a size of 51 nm in the egg water) and Ag^+^ ions (0.0649 LC_50_) in zebrafish embryos. The authors explained their observation as being a result of the increased surface energy and the quasi-stable state of the anisotropic nanoplates. However, we remain skeptical about the toxicity observed being a result of the shape of the NPs in the earlier study, owing to the size difference reported between the smaller nanoplates and the larger nanospheres. Another study [80] presented a concrete explanation for the greater toxicity of the Ag nanoplates (32 nm) (i.e., hitting 100% mortality of zebrafish embryos at a concentration of only 10 μg/mL) compared to that of spherical Ag NPs (10, 20, and 40 nm). The higher toxicity was attributed to the increased surface reactivity of Ag nanoplates due to their crystal defects (i.e., the irregular stacking planes of atoms in the crystal lattice). Excitingly, the authors concluded two facts: (i) the surface reactivity of the Ag nanoplates drives a different toxicity mechanism from that of the Ag nanospheres; and (ii) the refutation of the belief that the release of Ag^+^ ions from the surface of NPs is the main reason behind their toxicity, because even Ag nanoplates released fewer Ag^+^ ions than Ag nanospheres in Holtfreter’s media, and they induced greater toxicity. Sangabathuni and colleagues [81] avoided the size difference in investigating the shape-dependent toxicity of Au NPs by almost unifying the sizes of rod-shaped Au NPs (49 nm) and star-shaped Au NPs (46 nm) (as shown in Figure 5). They observed an initial accumulation (after 4 h) of rod Au NPs, followed by a clearance from adult zebrafish (injected intraperitoneally) after 24 and 48 h. In contrast, star Au NPs showed a steady cumulative state and prolonged sequestration (as shown in Figure 5). They attributed this discrepancy to the difference in the physical factors of the different shapes of NPs, such as the aspect ratio of rod Au NPs facilitating their rapid uptake and clearance, while the high friction coefficient of star Au NPs facilitated their slow clearance. Regarding NP surface charge, would it also impact NP toxicity? Truong et al. [82] revealed that Au NPs of the same size (1.5 nm) and concentration (50 μg/mL) had different impacts on the larval behavior of zebrafish based on the surface charge of their functional groups. Negatively charged 2-mercaptoethanesulfonic acid-Au NPs decreased the larval locomotor activity (i.e., distance swam by larvae) in the dark by 50% less than that of the neutral 2-(2-(2-mercaptoethoxy)ethoxy)ethanol-Au NPs, which were similar to the control group. Now the most conflicting question is what the main reason behind the toxicity of NPs is. Is it the Ag^+^ ions released from NPs? Or NPs themselves? Osborne et al. [83] demonstrated that Ag^+^ are the critical factor responsible for the toxicity of Ag NPs (35 nm, LC_50_ of 500 μg/L) in zebrafish embryos, especially at the gastrula stage (embryonic deformity at the 1 to 2 cell stage) with more expression of the heavy metal stress response gene (metallothionine 2) in this stage (i.e., 7.2 h post-fertilization, hpf) compared with after 24 hpf. This outcome is contrary to our previous results [84], which showed a greater toxicity (higher degree of mortality) to be induced by Ag NPs (9 nm) than by Ag^+^ in the form of AgNO_3_ due to the complexation of Ag^+^ with Cl^-^ when exposed in Holtfreter’s medium, decreasing the bioavailable exposure to Ag^+^. Moreover, the reported phenotypic toxicity of Ag NPs was entirely different from that of AgNO_3_ NPs. Ag NPs induced axial deformity, whereas AgNO_3_ induced deposition of Ag^+^ on the embryonic chorions. 

What could the administration of such NPs to humans induce? Human poisoning from Ag NPs and Ag^+^ ions has been demonstrated to be argyria (i.e., a blueish-grey discoloration of the skin induced by silver deposits). Additionally, the outcome of studies remains controversial with respect to Ag crossing the blood–brain barrier, and penetrating the extracellular fluid of the brain after oral administration. Other data refute the crossing of the blood–brain barrier by Ag [85]. A 62-year-old male was diagnosed with argyria with uniform blue-gray pigmentation on his head, neck, chest, and limbs. Within the sun-exposed region, conjunctiva, caruncle, and lunula of nails were also affected. The pigmented asymptomatic lesions developed within five years following weekly consumption of a bottle of AgNO_3_ by the patients after reading a publication describing the benefits of AgNO_3_ ‘in killing microorganisms’. The histopathological evaluation of the patient’s incisional biopsy demonstrated scattered extracellular black granules among collagen fibers that were also confirmed by electronic microscopy, showing the same dark brown pigmentation. The sun-exposed region’s discoloration was attributed to the photoactivation and metal reduction of the large amounts of Ag in such areas [86]. Moreover, even occupational exposure to NPs increases the systemic acute phase response. This is a long-term response for insoluble NPs, corresponding to a 45-year work-life [47]. These concerns regarding occupational exposure were highlighted in a previous case study, which demonstrated the high exposure level of apprentice welders to ultrafine particles (UFPs, with a size of 50 to 214 nm, with a majority of particles < 100 nm) that could ultimately result in the development of respiratory symptoms. UFP exposures and particle sizes were influenced by the type of welding process and welded metal and the time elapsed from the previous final welding [87]. Even though the focus of the present manuscript is on anti-CoV plasmonic NPs and QDs, titanium dioxide (TiO_2_) NPs remain one of the most widely used NPs, can rapidly penetrate the skin, and are poorly eliminated, inducing severe skin damage [88]. Monsé and co-workers [89] investigated the effect of inhaling airborne ZnO NPs in sixteen healthy nonsmoking volunteers for 4 h. ZnO NP concentrations of 0.5, 1, and 2 mg/m^3^ and sizes of 48, 63, and 86 nm, respectively, demonstrated significant concentration-dependent increases in acute phase proteins (CRP and SAA) and neutrophils in the blood of subjects exposed to concentrations of 1 and 2 mg/m^3^ of ZnO NPs. The authors then proposed a level of 0.5 to 1 mg/m^3^ ZnO NP to describe the No Effect Exposure Level (NOEL). Ultimately, we urge the scientific community to undertake further investigations, disclosing the possible adverse effects of different plasmonic NPs and QDs on human bodies.

## 4. What Safety Challenges Do Anti-CoV NPs Present?

Despite the tremendous number of publications dedicated to investigating the ecotoxicological effects of NPs on humans or in animals or cell cultures, most of these studies do not offer clear-cut statements on the safety of NPs. Conversely, they are mostly contradictory or mechanistic studies rather than toxicological ones [90]. A potential problem concerning NP toxicity is the multiple factors determining the NP toxicity, including chemical composition, particle size, shape, surface charge, and other physicochemical properties [45], for example, the thorough examination of spherical Ag NPs at the expense of other shapes overlooked by researchers. This spherical Ag NP shape almost lacks crystal defects that could be present in the understudied Ag nanoplates [80]. This problem is also exacerbated by the different synthesis procedures, raw materials, and reaction criteria needed to produce adequate volumes of uniform NPs with reproducible properties obtained across diverse research groups [42]. This irreproducibility extends to complex nanobiotechnology systems, raising the conflict within the literature to unprecedented levels [91]. To complicate the concerns surround NP safety, there is not even consensus regarding the assessment measurements concerning human and occupational exposure to NPs and UFPs [87]. Other general drawbacks of vaccines utilizing nanotechnology include the challenging cold chain requirements, which constitute a significant financial hurdle to the availability of such life-saving vaccines in developing nations [35]. 

Despite the tremendous amount of research conducted on the formation of NP-PCs, most of these studies have only been executed in vitro, failing to represent the complex in vivo environment, which constitutes a challenge to the capture of NPs after their administration in vivo. Another challenge that strikes at the heart of the NP-PC debate is the dynamic nature of physiological fluids (e.g., the flow of blood at different velocities, ranging from a few μm/s in capillaries to 60 cm/s in the ascending aortas), which elicits shear stress on NPs and results in new biomolecules [52]. Furthermore, far too little information and attention have been paid to the soft PC compared to the massive extent of investigations into hard PC [55]. An ironic fact is that proteins are not the sole biological entity interacting with NPs. The binding of lipids, such as plasma lipoproteins on NPs, has also been discussed in the literature [55]. To date, there is little agreement on the in vitro and in vivo toxicity findings for QDs, which could be explained by the precise dosages warranted under cultural conditions and the varying dosages maintained in vivo [48]. Another perceived problem with investigating the biological effects of NPs is their particle agglomeration under exposure conditions [80]. To date, there remains a lack of consensus on basic laboratory protocols within the infant nanotoxicology field [57].

On the one hand, assays for CRP are commercially available and commonly used as systemic inflammation biomarkers. On the other hand, these assays are fraught with caveats, because many factors influence the CRP levels, including body mass index, smoking, chronic inflammatory diseases, anti-inflammatory drugs, and infections [47]. For example, elevated CRP levels support the differential diagnosis of acute bacterial infections [92]. Recent work by the Wuhan team [93] concluded that serum levels of CRP could be used independently to assess and predict the severity of COVID-19. Among the 140 investigated COVID-19 patients, 91 (65%) patients showed increased CRP levels, and these were more significant in the severe group than the mild group. The authors drew a roadmap, indicating that patients with CRP > 41.8 mg/L were more prone to developing severe COVID-19 illness.

It is prudent to choose a suitable animal model for the investigation of emerging anti-CoV drugs. One reason behind the importance of animal model selection is that a small animal, for example, BALB/c and C57BL/6 mice, golden Syrian hamsters, and ferrets, could be infected with SARS-CoV without showing the disease clinically. Additionally, small BALB/c mice and golden Syrian hamsters are not susceptible to MERS-CoV infection. Rhesus macaques, a non-human primate, only develop self-limiting disease. By contrast, the most appropriate animal models for testing anti-SARS-CoV technologies are transgenic mice expressing human ACE2. The chief constraint remains the limited availability of these ACE2-transgenic mice [1]. With respect to SARS-CoV-2 specifically, the spike (S) glycoprotein includes the surface unit S1, which binds to ACE2 receptors. The transmembrane unit S2 is cleaved by human transmembrane serine proteases TMPRSS1 and TMPTSS2, making the co-expression of ACE2 and TMPRSSs crucial for SARS-CoV-2 infection. However, rats, mice, rabbits, and guinea pigs lack ACE2 receptors. In contrast, non-human primates, Syrian hamsters, ferrets, cats, and engineered chimeras, can mimic human infections and are promising animal models of SARS-CoV-2 infections. The challenges lie in the additional ethical justifications required to deal with non-human primate animal models, the scarcity of biosafety level III facilities, and the lack of trained personnel for executing such experiments [94]. 

## 5. Our Inputs: What Next?

A recent study [95] attributed the main reason behind severe COVID-19 to the progressive damage of lung epithelial–endothelial barriers, facilitating wide viral dissemination and spread. This endothelial damage could stem from the heightened immune responses mediated via complement activation, antibody-dependent enhancement, and cytokine release. The authors then highlighted the necessity of identifying COVID-19 patients in the early infectious phases and testing antiviral therapies at these phases to prevent viral entry and replication. Therefore, we believe that the integration of antiviral nanotheranostics into facemasks would achieve two goals. Firstly, they would inactivate viral particles, preventing their entry into the upper respiratory tract and avoiding even early phases of infections. Secondly, the safety issues associated with the nanoparticles involved would be reduced, as they are integrated within the facemasks. However, studies should be executed to show the absence of NP or ion leaching from the masks and investigate the consequence of NP inhalation from the masks into the lungs.

Regrettably, several intriguing treatment strategies failed to reach evaluation or registration after clinical trials during the previous SARS-CoV-1 and MERS-CoV outbreaks because of delays and the declining number of infected cases [2]. Furthermore, the host immune response to infection activates the coagulation cascade, where thrombin receptors (i.e., proteinase-activated receptors, PARs, chiefly PAR-1) facilitate an entwined relationship between coagulation, inflammatory, and fibrotic responses. This relationship shapes the lung pathology associated with COVID-19 [3]. Consequently, suppressing the excessive inflammatory response triggered by COVID-19 could have an invaluable impact on decreasing the death toll of severely ill patients [96]. The harmful effects of cytokine storms on the poor prognosis of COVID-19 call for other treatment methodologies targeting the cytokine storm, such as artificial-liver blood purification [96]. Even though concerns still revolve around the possible complications of steroids when treating COVID-19 patients, it makes no sense to delay administering a widely available and affordable drug, demonstrating reduced mortalities, especially in ventilated COVID-19 patients [97]. For other repurposed drugs, the FDA released a letter on 10 April 2020 [98] warning the public against self-medication using ivermectin products intended for treating animals (i.e., against heartworm diseases and certain parasites). The reason behind this concern is that the FDA has only assessed their safety and effectiveness in the intended animal species. Therefore, people should not use animal medications, because they could be hazardous to humans, unless an ivermectin form has been prescribed for people by a licensed health care provider and is obtained via a legitimate channel.

Regarding the human–animal interface of SARS-CoV-2 outbreaks, on the one hand, the reason behind the efficient SARS-CoV-2 spread between mink farms could be as simple as the high stocking animal densities on such farms. On the other hand, the in-depth reasons behind this efficient spread remain ambiguous, making it difficult to stop the outbreak among farms. A possible explanation for this rapid viral spread is that it is transmitted through infected humans, fomites, infectious animal droplets, or contaminated dust [13]. However, the implementation of strict preventative measures in the Netherlands did not stop the outbreak. Therefore, other reasons, such as wild animals and wind, should also be considered. Regardless of the transmission route, halting the spread between farms could be tricky because of the difficulty to control the movement of wild animals and the dissemination of asymptomatic human cases. Furthermore, farming conditions, including many different animals, constitute a risk of forming virus reservoirs and new mutations, as well as the spread of the virus into communities from farms through humans or escaped mink. Several other animals could also be SARS-CoV-2 infected, including raccoon dogs that, just like mink, are bred in farms (in a few countries) and live in the wild close to humans, even though no similar outbreaks have been detected among them yet. As it seems a daunting task to fully control the viral spread among companion animals, another hurdle is presented by limited resources, preventing the comprehensive administration of vaccines to animals, despite the importance of vaccinating susceptible animals (i.e., minks, cats, and zoo animals) [21]. We stress the necessity of regular, comprehensive animal surveillance and monitoring, specifically for minks, raccoon dogs, cats, and zoo animals, to avoid and predict SARS-CoV-2 human–animal and animal–human transmissions. Moreover, we agree with systemic genotyping recommendations and sharing the genome sequences of isolated SARS-CoV-2 strains from all infected animals to rapidly identify possible clusters and related variants. The world is now in a situation where One Health preparedness and response strategies are a must to synchronize efforts from different sectors (i.e., human health, animal health, and agriculture) in order to achieve a timely control strategy against the pandemic [12]. Additionally, we recommend further investigations on the UK and South African variants. Such further investigations would elucidate the role of mutations on several levels, including (i) the increased SARS-CoV-2 transmissibility, (ii) their effect on the potency of vaccine candidates, and (iii) the possibility of SARS-CoV-2 re-infectivity on both previously confirmed COVID-19 patients and vaccinated individuals. 

To provide elucidative clues on the nebulous toxicity of NPs and the correlation between their size and the elicited toxicity, the nanotoxicology community should develop a roadmap for standardized and harmonized toxicological testing procedures and methods for NPs [57]. Defining the affinity, stoichiometry, kinetics, and concentrations of NPs is a must for predicting their specific interactions with proteins to efficiently administer anti-coronavirus NPs in delivery systems. The dilemma remains in describing the contribution of PCs to ultimate distribution of such nanosystems in vivo [53]. Computational models using standard languages could also serve the prediction of NP toxicities [57] and help understand the impact of PC structure and fingerprints (protein patterns of NPs characterizing their PC) on cellular uptake and toxicity [52]. Development of research monitoring for airborne NP exposure (i.e., aerosol samplers in workplaces and environments), detectors for waterborne NPs, and sensors for measuring both exposure and potential hazards (e.g., production of reactive oxygen species) under different circumstances could robustly contribute to unveiling the impact of NPs on humans and the environment [43].

Several routes could mitigate the toxicity of NPs, including (i) inducing the aggregation of NPs using natural NPs that aggregate and immobilize engineered NPs at disposal sites. (ii) Surface coating of NPs using stable, environmentally friendly, or degradable coatings to avoid the adverse biological effects of NPs and their dissolution into toxic ions. (iii) Modifying the surface charge of NPs (e.g., using layer-by-layer coatings of polyelectrolytes) to reduce the cellular uptake and toxicity of NPs [51]. High-throughput approaches, such as the quantitative structure–activity relation (QSAR, describing the correlations between the physicochemical properties and bioactivity of NPs), are perceived as promising tools for elaborating the interactions at the nano–bio interface and embrace the assumption that NPs with correlated physicochemical properties induce similar bioresponses [52]. In the same vein, a previous report focusing on the toxicity of metallic NPs in zebrafish embryos concluded that the size of the NPs was as crucial in determining the toxicity of NPs as their chemical composition [45]. We refute this notion, because all different factors that induce different physicochemical properties of NPs influence the final toxicities of NPs. We believe that the size of NPs is a dominant feature in determining the toxicity issues of nanotheranostics and the antimicrobial properties of NPs [84,99]. Consequently, we recommend that any study implemented to study the toxicity issues of one of the physicochemical properties of NPs (e.g., the shape of NPs) should render all of the other physicochemical properties uniform (e.g., size, surface charge, and purity and chemical composition of NPs) and control the experimental procedures precisely in order to be able to draw concrete conclusions and perform a faithful elaboration regarding toxicity concerns. This is crucial, because any minor changes in the properties of NPs could induce large differences in the biological responses [60]. 

Elucidating the safety of NPs and NP formulations would result in a great increase in faith, maximizing public confidence and the reliable commercialization of such NPs or NP formulations. Nanoinformatics, considering FAIR (Findable, Accessible, Re-usable, and Interoperable) data principles, ensure nanosafety assessment. Nanoinformatics would achieve this assessment by predicting NP properties, nano–bio interfaces, NP transformations, and biological impacts, together with in silico approaches, in order to ensure the safety of NPs [100]. Recent technologies, namely organs-on-a-chip, mimic the in vivo environments and biological responses of entire organs via multichannel 3D microfluidic cell culture chips, and could traverse the gap between in vitro and in vivo studies by deciphering the PC dilemma [52]. Five years ago, the group of Zumla [1] recommended the development of novel, broad-spectrum, pan-CoV antiviral drugs effective against different CoVs as a “holy grail” treatment strategy against emerging CoV infections. Nowadays, dreams are coming true, and BNT162b2 and mRNA-1273 nanoformulation vaccines are close to receiving approval from the US Food and Drug Administration for human use [36]. During the final stages of submitting this manuscript, the vaccine had already been approved. After receiving the vaccination, the question is, for how long w the vaccine remain potent? What are the accurate percentages surrounding possible re-infection after being vaccinated? Would developing countries receive a sufficient share of the vaccine, moneywise and storage-wise? Some outside-the-box solutions for the required cold chain hurdle have been proposed, including vaccine delivery platforms and devices overcoming such hindrances in order to make COVID-19 vaccines equally available for developed and developing nations [35]. Additionally, some endeavors for decreasing the overwhelmingly high costs of cold shipment chains (reaching almost 80% of the total cost of vaccine development) have been undertaken through the collaboration of Suzhou Abogen Biosciences, Walvax Biotechnology, and People’s Liberation Army Academy of Military Sciences in China for COVID-19, developing a thermostable mRNA nano-vaccine. This vaccine candidate (i.e., ARCoV) is formed using the vesicle method, encapsulating the mRNA encoding the RBD of SARS-CoV-2 S glycoprotein in the lipid NPs [34]. What remains a worrying concern is the safety issues of NP formulations, as the whole nanotoxicity field is still in its early stages. Many research gaps need to be filled before using nanomedicine in comprehensive administrations in order to understand and predict the consequences of NPs scientifically and to guarantee an end to reasonably escalating public concern. We enthusiastically recommend engagement between multidisciplinary fields of virology, medicine, pharmacology, chemistry, nanotechnology, and toxicology, together with the industrial sectors under the umbrella of the WHO, to provide scientific, credible, and commercially and publicly transparent data on the consequences of administering anti-coronavirus NP formulations.

## 6. Conclusions

The present manuscript sheds significant light on the recent discoveries associated with SARS-CoV-2. We highlight the new SARS-CoV-2 variants, new cell entry mechanisms via neuropilin-1 co-factor, and olfactory transmucosal route to the CNS, inducing neurological symptoms and explaining the loss of taste and smell associated with SARS-CoV-2. We spot the overlooked SARS-CoV-2 human–animal and animal–human transmission areas to further exclude a saddening wide animal culling. Instead, we suggest parallel vaccination strategies for humans, minks, raccoon dogs, cats, and zoo animals using the NP formulations of the BNT162b2 and mRNA-1273 vaccines. We call for further studies on the UK and South African variants to unravel the role of mutations in transmissibility, vaccine candidate efficacy, and the re-infectivity of SARS-CoV-2.

We express our skeptical opinion regarding the comprehensive administration of such antiviral nanotheranostics in facemasks and NP formulations because of reported toxicities and the different NP parameters that need be closely studied and tightly controlled to avoid resulting biological toxicity. We raise an alarm bell with respect to the NPs employed in antiviral nanotheranostics, as more studies in multidisciplinary fields should be executed in order to clarify the odds surrounding infantile nanotoxicology for safe administration and public relief.

We suggest the administration of antiviral nanotheranostics on surfaces for the detection and self-inactivation of SARS-CoV-2, considering that investigations should first be performed on the toxicities of such systems on skin cells as a paradigm representing the contact of skin with surfaces containing anti-coronavirus NPs. This hot topic is currently under investigation in our experimental research agenda.

## Abbreviations

Angiotensin-converting enzyme 2	ACE2
C-reactive protein	CRP
cationic amphiphilic drugs	CADs
central nervous system	CNS
coronaviruses	CoVs
gold nanoparticles	GNPs
hard PC	HPC
hours post-fertilization	hpf
median lethal concentration	LC_50_
Middle East respiratory syndrome coronavirus	MERS-CoV
neuropilin-1	NRP1
nuclear factor kappa-light-chain-enhancer of activated B cells	NF-kB
open reading frames	ORFs
protein corona	PC
proteinase-activated receptors	PARs
quantitative structure-activity relation	QSAR
quantum dots	QDs
receptor-binding domain	RBD
SARS-related CoVs	SARSr-CoVs
serum Amyloid A	SAA
severe acute respiratory syndrome coronavirus 2	SARS-CoV-2
small airway epithelial cells	SAECs
soft PC	SPC
titanium dioxide	TiO_2_
ultrafine particles	UFPs
